# Substance-Specific Differences in Human Electroencephalographic Burst Suppression Patterns

**DOI:** 10.3389/fnhum.2018.00368

**Published:** 2018-09-21

**Authors:** Antonia Fleischmann, Stefanie Pilge, Tobias Kiel, Stephan Kratzer, Gerhard Schneider, Matthias Kreuzer

**Affiliations:** Department of Anesthesiology and Intensive Care, Klinikum Rechts der Isar, Technische Universität München, Munich, Germany

**Keywords:** burst suppression, anesthesia, general, electroencephalography, anesthetic monitoring, anesthetics, EEG patterns, humans

## Abstract

Different anesthetic agents induce burst suppression in the electroencephalogram (EEG) at very deep levels of general anesthesia. EEG burst suppression has been identified to be a risk factor for postoperative delirium (POD). EEG based automated detection algorithms are used to detect burst suppression patterns during general anesthesia and a burst suppression ratio (BSR) is calculated. Unfortunately, applied algorithms do not give information as precisely as suggested, often resulting in an underestimation of the patients’ burst suppression level. Additional knowledge of substance-specific burst suppression patterns could be of great importance to improve the ability of EEG based monitors to detect burst suppression. In a re-analysis of EEG recordings obtained from a previous study, we analyzed EEG data of 45 patients undergoing elective surgery under general anesthesia. The patients were anesthetized with sevoflurane, isoflurane or propofol (*n* = 15, for each group). After skin incision, the used agent was titrated to a level when burst suppression occurred. In a visual analysis of the EEG, blinded to the used anesthetic agent, we included the first distinct burst in our analysis. To avoid bias through changing EEG dynamics throughout the burst, we only focused on the first 2 s of the burst. These episodes were analyzed using the power spectral density (PSD) and normalized PSD, the absolute burst amplitude and absolute burst slope, as well as permutation entropy (PeEn). Our results show significant substance-specific differences in the architecture of the burst. Volatile-induced bursts showed higher burst amplitudes and higher burst power. Propofol-induced bursts had significantly higher relative power in the EEG alpha-range. Further, isoflurane-induced bursts had the steepest burst slopes. We can present the first systematic comparison of substance-specific burst characteristics during anesthesia. Previous observations, mostly derived from animal studies, pointing out the substance-specific differences in bursting behavior, concur with our findings. Our findings of substance-specific EEG characteristics can provide information to help improve automated burst suppression detection in monitoring devices. More specific detection of burst suppression may be helpful to reduce excessive EEG effects of anesthesia and therefore the incidence of adverse outcomes such as POD.

## Introduction

Burst suppression is a pattern of neuronal network activity that is characteristically seen in a highly inactivated brain, observed in a range of conditions such as hypothermia (Stecker et al., 2001), coma (Young, 2000), and in deep levels of general anesthesia (Rampil, 1998; Brown et al., 2010). In the electroencephalogram (EEG) burst suppression is marked by high voltage brain activity (bursts) and relatively low voltage activity (suppression; Rampil, 1998). Derbyshire et al. (1936) described different EEG patterns including burst suppression induced by the anesthetic avertin back in 1936. Thalamic cells that discharge synchronous rhythmic spikes to an otherwise unresponsive cortex seem to present a key mechanism of burst suppression (Steriade et al., 1994). Visual, somatosensory or auditory stimuli are capable of triggering bursts (Yli-Hankala et al., 1993; Hartikainen et al., 1995b; Hudetz and Imas, 2007). Kroeger and Amzica (2007) therefore considered burst suppression as a state of cortical hypersensitivity. Their results emphasized the absence of involvement of the autonomic nervous system, as no heart rate variations were recorded in response to the provocations. No large body of literature regarding the substance-specific differences of the EEG features during drug-induced burst suppression bursts does exist. Jäntti et al. (1993) found spindles to be characteristic for propofol induced burst suppression. The isoflurane bursts were described to have sharp waves, but not quite as sharp as the bursts in enflurane anesthesia. A very similar description has been made by Lipping et al. (1995) reporting the isoflurane bursts to have smoother waveforms compared to the very sharp enflurane spikes. Hartikainen et al. (1995b) studied the effects of isoflurane during burst suppression anesthesia. They found that with deeper states of anesthesia, suppression periods increase in duration while the total duration of bursts decreases (Hartikainen et al., 1995b). Most of the work regarding substance-specific differences comes from animal models. In 1996 Akrawi et al. (Akrawi et al., 1996) were first to describe substantial electrophysiological differences, in EEG burst suppression patterns, of different anesthetic agents in rats. They compared isoflurane, thiopental, etomidate and propofol at cortical and subcortical sites during burst suppression finding significant differences in EEG characteristics. Isoflurane showed the greatest peak-to-peak voltage and area under the curve (AUC), compared to the other three agents. For all agents, subcortical leads showed greater peak-to-peak voltage and AUC (a measurement for total power within bursts), compared to cortical leads. Differences were found in all agent pairs, except propofol and etomidate, both known to be GABA_A_ agonists. A comparison of isoflurane and I653 anesthesia (a volatile anesthetic structurally similar to enflurane) in pigs led to the conclusion that EEG patterns were similar at equipotent concentration (Rampil et al., 1988). Another animal study (Murrell et al., 2008) analyzing the burst suppression ratio (BSR) of various volatile anesthetic agents in rats, pointed out that isoflurane, sevoflurane and desflurane all cause burst suppression at concentrations necessary to provide surgical anesthesia. On the other hand, this study did not show suppression at any halothane concentration. Results from a more recent investigation in chicken show that burst suppression can occur at halothane MAC levels ≥2 (Mcilhone et al., 2018). Kenny et al. (2014) showed, in rats, that propofol and sevoflurane produce distinct burst suppression patterns. The duration, the peak-to-peak amplitude and the power of the sevoflurane-induced bursts were significantly greater than the propofol-induced bursts. Results from another study describe propofol burst suppression as smooth wave and isoflurane bursts as a clear on-off pattern, between bursts and suppression. The amplitudes during isoflurane bursts were significantly higher than the ones in propofol bursts (Hartikainen et al., 1995a). These results stem from experiments in rabbits and the authors highlight the general difficulty of translating findings from animal models to humans. These described differences show that substance-specific bursts have intrinsic EEG features. Nevertheless, current EEG-based monitoring systems that evaluate the hypnotic component in a patient of anesthesia by displaying an index only use very coarse algorithms for burst suppression (BS) detection.

They calculate an index—(B)SR—that indicates the occurrence and intensity of burst suppression defined as the duration of suppression periods. This information as well as the displayed EEG trace help to identify burst suppression patterns. This identification is important, because burst suppression seems to be an independent predictor for postoperative delirium (POD; Radtke et al., 2013; Fritz et al., 2016), a complication of general anesthesia frequently observed in elderly patients. The use of EEG-based monitors like the bispectral index (BIS, Medtronic, Dublin, Ireland) can help to titrate anesthetic agents to adequate levels of anesthesia and help to prevent unnecessarily deep levels with burst suppression and possible neurotoxic effects (Fedorow and Grocott, 2010). Nevertheless, adequate automatic detection of burst suppression does not seem straightforward (Palanca et al., 2009; Muhlhofer et al., 2017). A possible difference in substance-specific characteristics in the burst EEG may add to these difficulties. Such differences have been reported for animal models (Hartikainen et al., 1995b; Akrawi et al., 1996; Murrell et al., 2008; Kenny et al., 2014), and here we present findings from a patient study with controlled navigation to EEG burst suppression.

## Materials and Methods

### Study Design

We analyzed data from a previous clinical study conducted to evaluate the EEG and cerebral state index (CSI) characteristics at different levels of general anesthesia. In short, the CSI is an unitless index that inversely correlates to a patient’s level of consciousness. A CSI of 90–100 for instance reflects a fully awake patient, and a range between 40 and 60 is considered an adequate range to perform surgery. The publication by Jensen et al. (2006) provides a very detailed description of the underlying algorithms. Our study was carried out in accordance with the recommendations of the “Ethics Committee of Technical University of Munich, Munich, Germany” with written informed consent from all subjects in accordance with the Declaration of Helsinki. The protocol was approved by the “Ethics Committee of the Technische Universität München, Munich, Germany”, Ethical Committee N° 1239/05. After informed written consent to the study, 45 adult patients were included undergoing elective surgery under general anesthesia with an American Society of Anesthesiologists physical status I or II. Exclusion criteria were neurological or psychiatric diseases in the past, medications affecting the central nervous system, alcohol or drug abuse, and the indication of a rapid sequence induction (e.g., pregnancy, emergency).

The patients were assigned to three different study groups (sevoflurane group, isoflurane group, propofol group, *n* = 15 each), chosen by the anesthetist in charge. A randomization was deliberately abandoned, to reflect clinical daily routine. Sufentanil was administered as analgesic for the isoflurane and sevoflurane group and remifentanil for the propofol group. Atracurium or mivacurium were applied as neuromuscular blocking agents. Anesthesia was slowly induced with intravenous injections of propofol. After tracheal intubation, propofol, sevoflurane and isoflurane were administered according to clinical practice. After skin incision, anesthetic depth was increased until burst suppression pattern appeared in the EEG. Burst suppression was identified by the first episode of suppression with a length of at least 3 s. Thereafter, a return to baseline (general anesthesia adequate for surgical procedure) was performed by decreasing anesthetic depth.

### EEG Recording

The used EEG traces were recorded at a sample rate of *f*_s_ = 100 Hz using the cerebral state monitor (CSM) Link software (Danmeter, Odense, Denmark). Electrode positions were according to the manufacturer recommendation with one electrode placed on the central forehead, another on the left mastoid, and the third electrode on the left side of the forehead. The recommended electrode placements is described in the manual for the CSI^1^. Recorded EEG, the CSI, as well as the processed BSR were stored in a .csv file.

### Burst Suppression Selection

**Figure 1:**
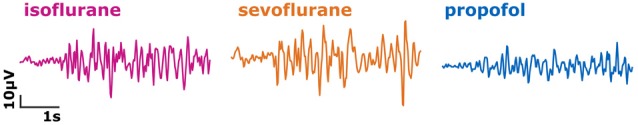
Representative bursts for isoflurane (left, purple), sevoflurane (middle, orange) and propofol (right, blue).

The selection of the first burst after a suppression episode was based on visual inspection. To the researcher, the anesthetic agent used in the single recordings was blinded during the selection of bursts. The first distinct burst identified in the EEG was cut out for further analysis. BSR and CSI (generated by the CSM) provided guidance in finding the first burst. A burst was defined as a sharp increase in amplitude and frequency following a period of suppression longer than 1 s, i.e., a “silent second” (Pilge et al., 2014) Initially the silent second presented a visual criterion to identify BS in an animal model (Korkmaz and Wahlström, 1997). If the very first burst was showing artifacts, the first clear burst without interference was selected. The selection was approved by an independent investigator. Figure 1 displays representative bursts for each anesthetic agent and Supplementary Figure S1 presents exemplary bursts inclusive the “silent second.”

### Burst Analysis

For the analysis we limited ourselves to the first 2 s of the burst. We decided on this approach to focus on substance-specific effects on EEG characteristics and not to bias our investigations by different burst lengths, endings of a burst that cannot be clearly identified and changing burst dynamics with ongoing burst duration.

#### Power Spectral Density

We calculated the power spectral density (PSD) of the burst episode using the MATLAB *pwelch* function with default settings and the NFFT set to 128, resulting in a frequency resolution of 0.78 Hz. We obtained the normalized PSD (nPSD) by dividing the power at each frequency by the sum of power between 6.25 Hz and 30.47 Hz. We chose this normalization interval because of the CSM cutoff frequency of the high pass at 6 Hz. The 30.47 Hz limit is arbitrary, but based on published findings that suggest EEG frequencies below 30 Hz mainly reflect cortical activity and higher frequencies may be increasingly contaminated by EMG (Greif et al., 2002; Bonhomme and Hans, 2007). The decimal places are because of the frequency bins constructed by the *pwelch* function. We dismissed the first 100 ms of burst onset to make sure we did not include any suppression.

#### Amplitude and Slope Analysis

In order to evaluate the absolute amplitudes during the first 2 s of the first burst, we evaluated the 99th percentile of the absolute amplitudes. We chose the percentile approach to add some robustness to our analyses. A similar percentile approach was used to analyze REM sleep episodes (Silvani et al., 2017). In order to evaluate the slope, i.e., the amplitude change over time, we calculated the first derivative of the EEG and defined the 99th percentile absolute value of the derivative as absolute slope.

#### Permutation Entropy

We further calculated the permutation entropy (PeEn; Bandt and Pompe, 2002), an ordinal time-domain parameter. PeEn has been used to evaluate different levels of general anesthesia and showed superior results when compared to other (spectral) approaches (Jordan et al., 2008; Olofsen et al., 2008) Essentially, entropic measures like PeEn present a signal analytical approach to evaluate EEG features in the time domain. Recent research revealed that PeEn seems to function as a proxy for EEG oscillation characteristics (Berger et al., 2017). PeEn evaluates the probability distribution of ordinal rank patterns of length *m*. Considering our short EEG segments, we defined the embedding dimension *m* = 3 and the time lag *τ* = 1, parameter settings that were commonly used for EEG analysis (Jordan et al., 2008; Olofsen et al., 2008).

### Statistical Analysis

#### Demographics

We analyzed the demographic data with MATLAB using the Kruskal-Wallis test with a *post hoc* Dunn’s test for multiple comparisons for age, weight and height. For evaluation of differences in sex and ASA status we used the Freeman-Halton extension of the Fisher exact test using an online source^2^.

#### EEG Analysis

In order to evaluate possible substance-specific effects on EEG burst features, we used a series of statistical approaches. Therefore, we used the first 2 s of EEG of the first burst of each patient.

For the descriptive statistics, we decided to present the median and median absolute deviation or the median and the single experiments. For the evaluation of differences in the spectral power features we calculated the AUC of the receiver-operating characteristic and 10k-fold bootstrapped 95% confidence intervals (CIs) using the MATLAB-based MES toolbox (Hentschke and Stüttgen, 2011). We chose this approach because of its nonparametric nature. In general, it is related to a Wilcoxon statistic (Jordan et al., 2010). Following studies using a similar approach with a different test we report only results as significant when neighboring frequencies showed significant differences (Akeju et al., 2014). We considered a difference between two distributions significant, if the 95% CI did not contain 0.5. We further decided to indicate AUC > 0.7 that depict a fair and relevant effect (Vivo and Franco, 2008). For analysis of differences in amplitude, slope, and PeEn we used the Kruskal-Wallis test with a *post hoc* Dunn’s test being the appropriate multiple comparison test (Elliott and Hynan, 2011). We used the *dunn* function for MATLAB (Cardillo, 2006). Additionally, we also calculated the AUC as a measure of effect size.

## Results

### Demographics

We did not observe a significant difference in the distribution of age, size, sex, and ASA status among the groups. The patients undergoing isoflurane anesthesia had a significant higher body weight (*p* < 0.05; Dunn’s test) than the patients in the sevoflurane and propofol group, but the body mass index (BMI) was not significantly different among the groups. Table 1 contains the detailed information regarding the demographics. Further, 13 out of 15 patients received benzodiazepines for oral premedication according to standard clinical practice shortly before they were transported to the operation theatre: they primarily received 3.75–7.5 mg midazolam or in rare cases 10–20 mg clorazepate.

**Table 1:** Demographic data of the three patient groups; Kruskal-Wallis and Freeman-Halton test for multiple comparison of age, size, weight, gender and ASA status.

Group	Propofol	Sevoflurane	Isoflurane	Kruskal-Wallis
Age (years)	57 [45 67]	43 [38.5 63.5]	40 [33 46.5]	*p* = 0.1008
Size (cm)	168 [161 178.5]	173 [167 179]	180 [173 185.5]	*p* = 0.1059
Weight (kg)	71 [68 77]	75 [69.5 79.5]	90 [81 100]	*p* = 0.0021*
BMI	25.4 [23.2 31.2]	25.8 [23.8 36.6]	28.4 [25.8 34.1]	*p* = 0.1474
				*Freeman-Halton*
Sex (m/f)	7/8	9/6	11/4	*p* = 0.3296
ASA (I/II)	10/5	8/7	10/5	*p* = 0.6839

The data are presented as median with 1st and 3rd quartile in square brackets. *The Dunn’s test revealed a significantly (*p* < 0.05) higher weight in the isoflurane group.

### Power Spectral Density of the Bursts

**Figure 2:**
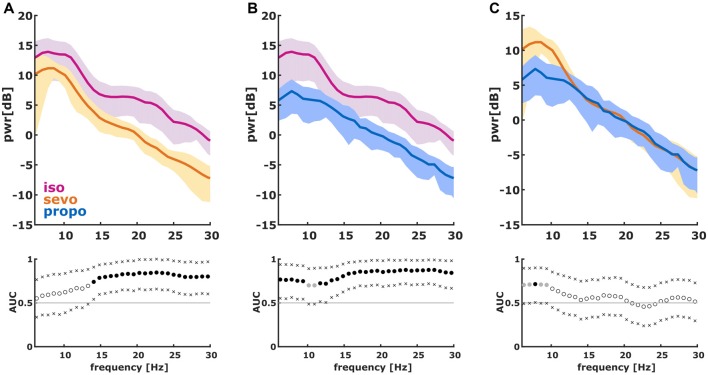
Power spectral density (PSD) in the 6–30 Hz range of the first 2 s of the first burst. (We dismissed the first 100 ms of burst onset to make sure we did not include any suppression). The solid lines present the median and the shaded areas the median absolute deviation for the comparisons of **(A)** isoflurane and sevoflurane, **(B)** isoflurane and propofol, and **(C)** sevoflurane and propofol. In the area under the curve (AUC) plots, a filled circle in black indicates significance and a gray circle indicates a non-significant AUC > 0.7. The non-filled circles indicate AUC < 0.7 with 95% confidence intervals (CIs) inclusive 0.5, i.e., there is no effect. The x indicate the upper and lower limits of the 95% Ci.

**Figure 3:**
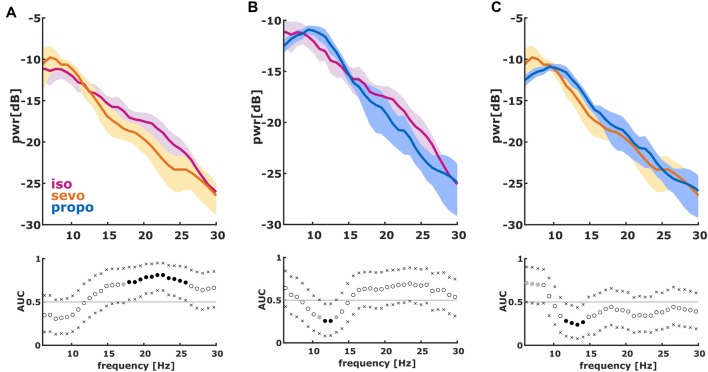
Normalized PSD (nPSD) in the 6–30 Hz range of the first 2 s of the first burst. (We dismissed the first 100 ms of burst onset to make sure we did not include any suppression). The solid lines present the median and the shaded areas the median absolute deviation for the comparisons of **(A)** isoflurane and sevoflurane, **(B)** isoflurane and propofol and **(C)** sevoflurane and propofol. In the AUC plots, a filled circle in black indicates significance and a gray circle indicates a non-significant AUC > 0.7. The non-filled circles indicate AUC < 0.7 with 95% CIs inclusive 0.5, i.e., there is no effect. The x indicate the upper and lower limits of the 95% Ci.

We found significant differences in the PSD and nPSD among substance-specific bursts. Isoflurane-induced bursts had higher power in the almost complete frequency range when compared to propofol-induced bursts. Compared to sevoflurane, isoflurane-induced bursts had higher power in the higher frequencies of ~14 Hz and more. Sevoflurane-induced bursts had potentially higher power in the lower frequencies from 6 Hz to ~10 Hz. In the analysis of nPSD, isoflurane-induced bursts maintained their higher power in frequencies corresponding to the EEG beta-range (i.e., ~12–25 Hz) when compared to sevoflurane. Propofol-induced bursts had higher normalized power in the ~12 Hz range than isoflurane- and sevoflurane-induced bursts. Sevoflurane-induced bursts had a lower frequency compared to propofol. Figure 2 presents the PSD plots and Figure 3 the nPSD plots together with corresponding AUC values with 95% CIs.

### Burst Analysis

#### Absolute Amplitude

**Figure 4:**
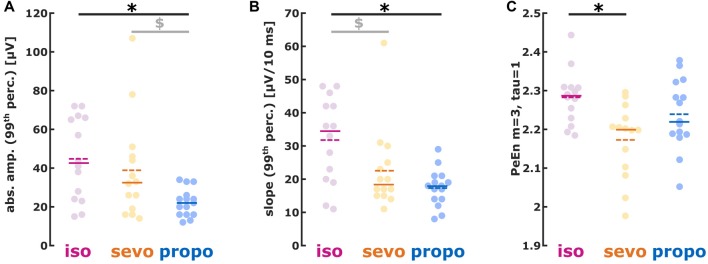
**(A)** Absolute amplitude, **(B)** first derivative (slope) and **(C)** permutation entropy (PeEn) of burst activity. **(A)** isoflurane- and sevoflurane-induced had higher amplitudes than propofol-induced bursts; **(B)** isoflurane-induced showed a steeper slope than sevoflurane- and propofol-induced bursts. **(C)** Sevoflurane-induced bursts had lower PeEn than isoflurane-induced bursts. *p < 0.05 after *post hoc* correction; ^$^AUC > 0.7 i.e., relevant effect with the 95% CI not containing 0.5, i.e., the effect being different from chance.

We found different absolute burst amplitudes (99th percentile) in a substance specific manner (Kruskal-Wallis: *p* = 0.0119, Chi-squared = 8.87) with lower amplitudes for propofol (isoflurane vs. propofol: *p* < 0.05 Dunn’s *post hoc*, AUC (95% CI): 0.81 (0.62 0.96); sevoflurane vs. propofol *p* > 0.05, AUC (95% CI): 0.72 (0.51 0.90)). Hence, AUC indicated a medium to strong effect on burst amplitude when comparing the volatiles to propofol (Figure 4A). Table 2 contains the median values together with the 1st and 3rd quartile of the absolute amplitude.

**Table 2:** Median values inclusive 1st and 3rd quartile for the absolute amplitude, the absolute slope, as well as the permutation entropy (PeEn) of the substance-specific bursting activity.

	Absolute amplitude (μV)	Absolute slope (μV/10 ms)	PeEn (*m* = 3, tau = 1)
Isoflurane	42.5 [24 66]	34.5 [20 42]	2.29 [2.23 2.31]
Sevoflurane	32.5 [19 46]	18.5 [15 25]	2.20 [2.10 2.23]
Propofol	22 [16 15.5]	18 [15 20.5]	2.22 [2.18 2.31]

#### Absolute Slope

The slope analysis revealed a steeper burst slope for isoflurane compared to sevoflurane and propofol (Kruskal-Wallis: *p* = 0.0102, Chi-squared = 9.18). Again, the AUC indicated substance-specific medium to strong effects on the burst slope (isoflurane vs. sevoflurane: *p* > 0.05, AUC (95% CI): 0.72 (0.51 0.91); isoflurane vs. propofol: *p* < 0.05, AUC (95% CI): 0.82 (0.64 0.97)). Table 2 contains the median values together with the 1st and 3rd quartile of the absolute slope and Figure 4B the single patients’ results.

#### Permutation Entropy

Isoflurane-induced bursts showed higher PeEn than sevoflurane-induced bursts. There were no differences in PeEn between isoflurane-and propofol-induced bursts as well as between sevoflurane- and propofol-induced bursts (Figure 4C; Kruskal-Wallis: *p* = 0.0153, Chi-squared = 8.63; isoflurane vs. sevoflurane: *p* < 0.05, AUC (95% CI): 0.83 (0.67 0.96)). Table 2 contains the median values together with the 1st and 3rd quartile of the PeEn analysis.

## Discussion

General anesthesia is defined as a drug-induced, reversible state of unconsciousness including amnesia, immobility and analgesia (Brown et al., 2010). As the level of anesthesia deepens, the EEG shows an increase in low-frequency, high-amplitude activity. Finally, at even higher doses of different volatile or intravenous anesthetics burst suppression can occur in the EEG (Brown et al., 2010). As described in the “Introduction” section, the knowledge regarding substance-specific differences in EEG features of BS bursts is rather sparse (Jäntti et al., 1993; Lipping et al., 1995), whereas there is some information from animal models (Rampil et al., 1988; Hartikainen et al., 1995a; Akrawi et al., 1996; Kenny et al., 2014). With our results we can add more information regarding substance-specific differences in humans and help to link some findings from animal models. We focused on the first 2 s of the first burst observed. This procedure ensures that we evaluated the burst right after the EEG (i.e., the state of the brain) switched for non-BS to BS patterns. This helps to overcome the fact that BS features change with concentration (Hartikainen et al., 1995b).

We also found, as has been described in animals (Akrawi et al., 1996; Kenny et al., 2014), that propofol bursts had the lowest amplitudes when compared to volatile anesthetics. Further, isoflurane-induced bursts in our study had the steepest slopes, while sevoflurane-induced bursts had the lowest PeEn indicative of a very regular signal. Another finding was the strong oscillatory component around 10 Hz in the propofol-induced bursts as determined by the nPSD analysis agreeing with the described spindles (Jäntti et al., 1993). In our analyses, the bursts induced by volatiles did not show this oscillatory component.

### Possible Mechanistic Description for the Differences in Burst Characteristics

Cortical burst suppression bursts seem to be mediated by an excitatory thalamic input to hyperexcitable cortical neurons (Kroeger and Amzica, 2007), i.e., the unresponsive cortex is captured by strong and synchronized thalamic activity (Steriade et al., 1994; Brown et al., 2010). They may also be triggered by glutamate-mediated excitatory transmission (Lukatch et al., 2005). Therefore, a substance-specific modulation of anatomic or molecular key structures within the thalamocortical network may account for the observed differences in bursts. Since the different anesthetics modulate substance-specific molecular targets that differ to some extent (Franks, 2008), the generated burst patterns can be different as well. Propofol seems to decrease thalamocortical excitatory neurotransmission by increased GABAergic inhibition of cortical pyramidal neurons in combination with an enhancement of the inhibitory input from the reticular thalamic nucleus to thalamocortical relay neurons (Koyanagi et al., 2014). However, results from *in vitro* experiments showed that propofol, in contrast to sevoflurane, does not decrease the intensity of cortical depolarization after thalamic stimulation (Kratzer et al., 2017). In the EEG, propofol leads to pronounced slow-wave and alpha-band activity during general anesthesia (Akeju et al., 2014). In our analyses, we could also observe a more pronounced alpha-oscillation in the propofol-induced bursts when compared to the volatiles. The volatile anesthetics, like isoflurane and sevoflurane, exert more unspecific effects on multiple neuronal targets. They enhance GABA- and glycinergic inhibition, impair excitatory neurotransmission and affect a variety of voltage-gated ion channels (Rudolph and Antkowiak, 2004). These different mode of actions between volatiles and propofol seem to account for differences in the EEG under general anesthesia (Akeju et al., 2014) and, as shown here under burst suppression. Another interesting result is the difference in burst EEG between isoflurane and sevoflurane, despite similar mechanisms of action. Burst duration and amplitude are sensitive to NMDA receptor antagonists, gap junction blockers, and extracellular calcium (Kroeger and Amzica, 2007). Isoflurane seems to inhibit NMDA receptors with a higher potency than sevoflurane (Solt et al., 2006). Hence, our observed differences in isoflurane- and sevoflurane induced bursts may arise from differential impacts on NMDA receptors, but it is too early to draw definitive conclusions.

### Impact of Our Study

Our results suggest that the described substance-specific differences occur in human EEG bursts in similar fashion. Isoflurane and sevoflurane bursts were of higher amplitude than propofol bursts. Isoflurane bursts had the steepest slopes, i.e., the strongest changes in amplitude within a short time. Sevoflurane in contrast seemed to trigger the most regular bursts as depicted by low PeEn. All of these findings are reflected in the PSD that indicate a higher general power in the bursts during volatile anesthesia. In contrast to sevoflurane, isoflurane-induced bursts showed more activity in the higher frequencies, a behavior also reflected in the lower PeEn of sevoflurane-induced bursts.

### Implications for Monitoring

With our study we could identify substance-specific differences in EEG burst patterns. These findings could help to optimize EEG-based “depth of anesthesia” monitoring at these very deep levels of general anesthesia. The BSR of the BIS detects suppression using an amplitude threshold of 0.5 μV (Rampil, 1998). For low index values indicating very deep anesthesia, the BSR is defining the BIS (Bruhn et al., 2000). A very similar, threshold-based detection-algorithm is part of the CSI (Jensen et al., 2006). The Entropy Module (GE Healthcare, Chicago, IL, USA), another EEG-based monitoring system that evaluates the hypnotic component of anesthesia (Viertiö-Oja et al., 2004), uses a nonlinear energy operator that is calculated from two different (slow/fast) EEG frequency bands (Särkelä et al., 2002). For the SEDLine patient state index (Masimo, Irvine, CA, USA), also a EEG-based system to evaluate the patients’ level of (un-)consciousness (Drover and Ortega, 2006), that burst detection algorithm is proprietary. We are confident that substance-specific burst detection, based on differences described in this article, may help to optimize monitoring. Scientific investigations also dealt with the automated detection of EEG burst suppression. Some of these approaches also use defined EEG thresholds (Chemali et al., 2013) or local signal variance (Brandon Westover et al., 2013; An et al., 2015), or higher order spectral analysis (Schack et al., 2001). Hence, substance-specific differences in EEG burst characteristics may also influence the performance of these classifiers. A number of limitations of automated machine-generated burst suppression detection were described in a study by Muhlhofer et al. (2017). The authors report that the automated burst suppression detection of the SEDLine significantly underestimated the real occurrence of burst suppression as identified through visual expert assessment. Furthermore, the neurologists’ consensus rating was significantly associated with the incidence of POD, while the relationship between the calculated SEDLine BSR and the incidence of POD was not significant. Our findings regarding substance-specific EEG burst features may help to develop better strategies to reliably catch these episodes in the recorded signal.

### Limitations

Of course, there are some limitations to our investigation. First of all, our EEG recordings do not contain the very low delta frequencies because of the intrinsic filter settings of the CSM device. Hence, we could not evaluate differences in these low frequencies. But other groups used similar frequency ranges for their burst suppression classification (Chemali et al., 2013). Because we used only single channel EEG recordings, we cannot describe any substance-specific differences in e.g., interhemispheric EEG synchrony. The frontal recording sites do not allow any speculations regarding differences in EEG burst patterns at other recording sites. These issues should all be part of future investigations. We also only focused on the initial 2 s of a burst and hence we do not draw any conclusions regarding burst length and changes in burst features with burst time. An EEG based monitor should be able to indicate the onset of burst suppression as soon as possible. This is important because both—incidence and increasing duration of EEG suppression—increase the risk of POD. Another issue we would like to mention and that possibly should be investigated in the future, is the difference of opioids used between the volatile anesthetic groups and the propofol group. Opioids in general may have some influence on burst suppression (Kortelainen et al., 2008). Further, benzodiazepines may have an effect on EEG activity: they increase beta (spindle) activity and reduce lower (alpha) frequency activity (Schulte am Esch and Kochs, 1990). Very high intravenous doses of benzodiazepines, e.g., as used for drug-induced coma in refractory status epilepticus, can lead to burst suppression EEG (Kang et al., 2015). In our study, we predominately used low oral doses of short-acting benzodiazepines. An additive sedative effect may be assumed but may have affected study patients in a comparable manner.

## Conclusion

Our findings describe substance-specific characteristics of EEG burst onset during burst suppression under general anesthesia. This new information can help to improve the reliable and fast routine detection of burst suppression and hence help to prevent unnecessarily deep levels of anesthesia.

## Author Contributions

MK designed and conducted analysis, wrote the manuscript. AF analyzed data and wrote the manuscript. TK and SP designed the study, conducted the study and helped to write the manuscript. SK helped to write the manuscript and provided critical feedback. GS helped to design the study, helped to write the manuscript and provided critical feedback.

## Conflict of Interest Statement

The authors declare that the research was conducted in the absence of any commercial or financial relationships that could be construed as a potential conflict of interest.

## Footnotes

^1^https://www.danmeter.dk/en/files/CSM-Monitor-MKII---Manual-561105003--US-only-.pdf

^2^http://vassarstats.net/fisher2x3.html

## Supplementary Material

FIGURE S1 The first burst after an initial silent second for three patients from each substance category each. The ivory boxes display the silent second and the 2 s of burst electroencephalogram (EEG) used for analysis. This 2 s approach was chosen because for monitoring purposes the burst onset is of significant interest. Further, with ongoing burst duration, the EEG features of the bursts change. Further, the bursts are of different length, within and between groups, but the offset detection is not straightforward.
