# Supplementary figures and images for: Substance-Specific Differences in Human Electroencephalographic Burst Suppression Patterns
Source: Front Hum Neurosci. 2018 Sep 21;12:368. doi: 10.3389/fnhum.2018.00368 (PMC6160564; doi:10.3389/fnhum.2018.00368)

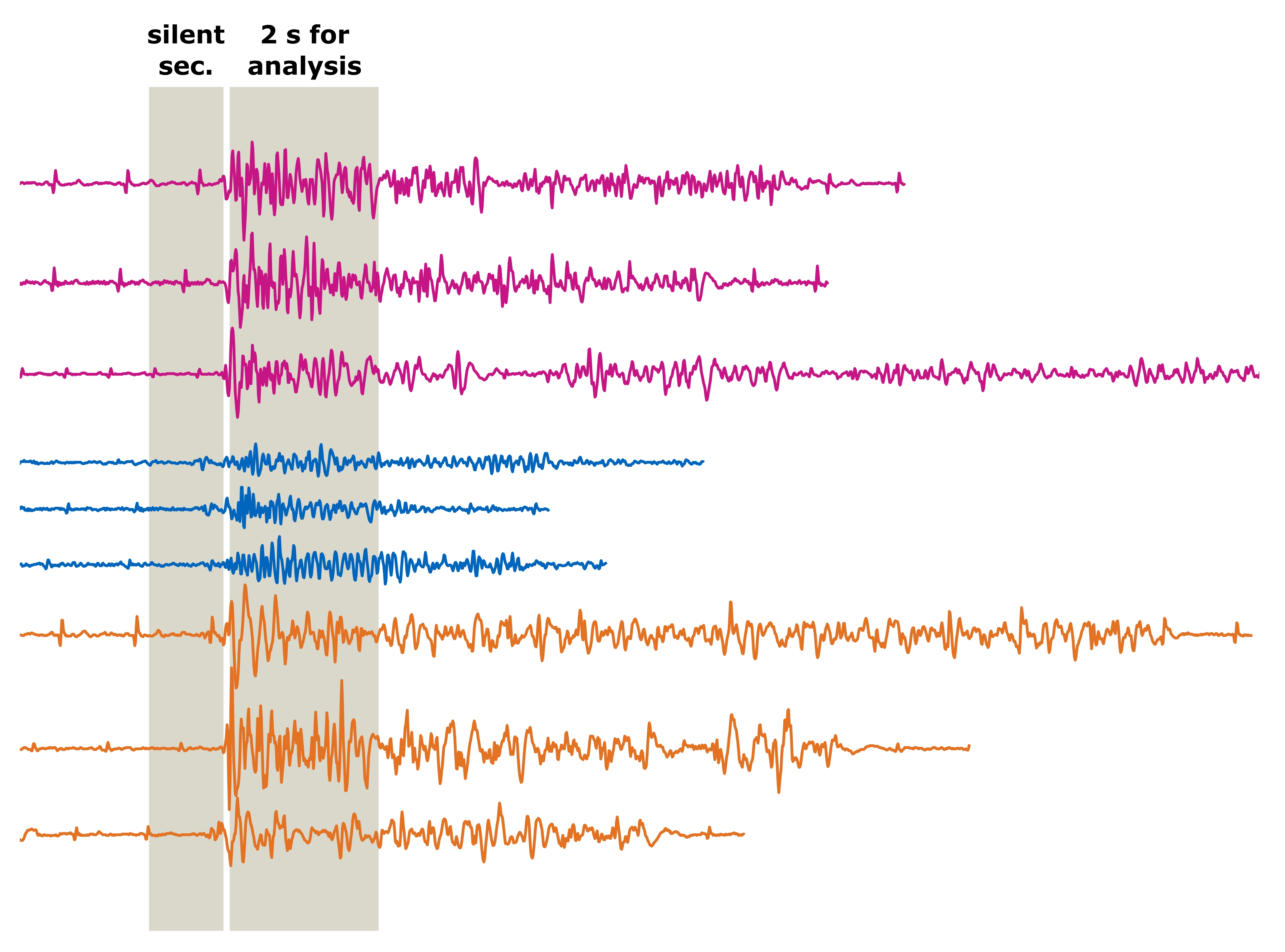

Supplement: FIGURE S1 — The first burst after an initial silent second for three patients from each substance category each. The ivory boxes display the silent second and the 2 s of burst electroencephalogram (EEG) used for analysis. This 2 s approach was chosen because for monitoring purposes the burst onset is of significant interest. Further, with ongoing burst duration, the EEG features of the bursts change. Further, the bursts are of different length, within and between groups, but the offset detection is not straightforward. [file Image_1.JPEG]
